# How Can People Be so Good at Intercepting Accelerating Objects if They Are so Poor at Visually Judging Acceleration?

**DOI:** 10.1177/2041669515624317

**Published:** 2016-01-27

**Authors:** Eli Brenner, Inés Abalo Rodriguez, Victor Estal Muñoz, Sabine Schootemeijer, Yannick Mahieu, Kirsten Veerkamp, Marit Zandbergen, Tim van der Zee, Jeroen BJ Smeets

**Affiliations:** Department of Human Movement Sciences, VU University, Amsterdam, The Netherlands

**Keywords:** Interception, catching, acceleration, motion, motor control, learning, vision

## Abstract

People are known to be very poor at visually judging acceleration. Yet, they are extremely proficient at intercepting balls that fall under gravitational acceleration. How is this possible? We previously found that people make systematic errors when trying to tap on targets that move with different constant accelerations or decelerations on interleaved trials. Here, we show that providing contextual information that indicates how the target will decelerate on the next trial does not reduce such errors. Such errors do rapidly diminish if the same deceleration is present on successive trials. After observing several targets move with a particular acceleration or deceleration without attempting to tap on them, participants tapped as if they had never experienced the acceleration or deceleration. Thus, people presumably deal with acceleration when catching or hitting a ball by compensating for the errors that they made on preceding attempts.

## Introduction

People are known to judge acceleration poorly and indirectly (Brouwer et al., 2002; Calderone & Kaiser, 1989; Gottsdanker, Frick, & Lockard, 1961; Watamaniuk & Heinen, 2003; Werkhoven, Snippe, & Toet, 1992). When interacting with moving objects, they ignore the moving objects’ accelerations for controlling various aspects of their movements (Benguigui & Bennett, 2010; Lee, Port, & Georgopoulos, 1997; Lee, Young, Reddish, Lough, & Clayton, 1983; Port, Lee, Dassonville, & Georgopoulos, 1997). In many of the cases in which acceleration is not evidently ignored, the apparent use of information about the object’s acceleration might just reflect the fact that movements are continuously adjusted on the basis of continuously updated estimates of the object’s position (Brenner & Smeets, 2011; Dubrowski & Carnahan, 2002) and velocity (Brenner, de Lussanet, & Smeets, 2002). When the acceleration of horizontally moving targets that people are trying to intercept is varied randomly across trials, people make systematic errors that correspond with ignoring the acceleration during the delay between when visual information reaches the eye and when the arm responds to such information (Brenner & Smeets, 2015). This is what one would expect if the fact that the target is accelerating is ignored, but the movement is constantly adjusted to account for the changes in target position and velocity that result from such acceleration.

Despite not being able to deal with acceleration reliably, people can time the way they hit falling balls very precisely (Brenner, Driesen, & Smeets, 2014; Brenner, van Dam, Berkhout, & Smeets, 2012; McLeod & Jenkins, 1991; McLeod, McLaughlin, & Nimmo-Smith, 1985). Moreover, they can intercept balls falling under gravitational acceleration with very little visual information (Katsumata & Russell, 2012; López-Moliner, Brenner, Louw, & Smeets, 2010; Zago, McIntyre, Senot, & Lacquaniti, 2008, 2009), and even if the ball initially had a lower acceleration because it was rolling down a slope (La Scaleia, Zago, & Lacquaniti, 2015). They even assume that there is some gravitational acceleration when visual information indicates that there is none (McIntyre, Zago, Berthoz, & Lacquaniti, 2001; Senot, Zago, Lacquaniti, & McIntyre, 2005; Zago et al., 2004), suggesting that performance is based on experience with gravity rather than on perceiving the gravitational acceleration.

In daily life, people encounter all sorts of accelerations, not only the acceleration of freely falling objects. Objects can slide or roll down slopes, in which case the acceleration by gravity depends on the slope, and there is deceleration by friction as well as air resistance (we use deceleration to refer to negative acceleration when describing specific cases, but when we refer to acceleration in general, we mean both acceleration and deceleration). Moreover, objects are often actively accelerated and decelerated, for instance, when other people hand them to you.

In many cases, one could have some estimate of the acceleration from experience. For instance, if one sees a ball rolling down a slope, one might remember how balls rolled down similar slopes in the past. Similarly, one will have experienced being given a glass many times before, and therefore, anticipate a certain deceleration as the glass approaches. One might not even just anticipate a certain constant acceleration but anticipate the same whole pattern of motion that one observed on similar or recent occasions. If people rely on experience to estimate how an object will move, rather than on instantaneous visual information, it would make sense to only consider experience under similar conditions, because there is no reason to expect the same pattern of motion for completely different objects or circumstances. One might remember a wide range of contexts with associated patterns of acceleration and pick the most likely pattern of acceleration on the basis of the context to guide one’s actions. Such a strategy could also account for people being able to anticipate how a virtual ball will bounce off a visible surface (Diaz, Cooper, Rothkopf, & Hayhoe, 2013) or how a dent or bump in a slope that the ball is rolling down will influence the ball’s motion (de Rugy, Marinovic, & Wallis, 2012).

An alternative is that people might not estimate the acceleration at all. Instead, they might just compensate for errors that arise from not considering the acceleration by adjusting their next movement in response to feedback (Brenner, Canal-Bruland, & van Beers, 2013). If an action is repeated over and over again, they might gradually adjust their behavior to the prevailing acceleration, without ever considering the fact that the errors had anything to do with acceleration. This can only help people cope with acceleration if the same acceleration is encountered repeatedly, and only if people receive feedback about their errors.

In the present study, we examined whether people learn to associate a certain pattern of acceleration with a certain context, or whether they only adjust their movements in response to recent feedback. We did so by asking participants to try to tap on virtual disks moving across a surface. We examined whether associating different target accelerations with different appearances of the surfaces (each suggesting a different coefficient of friction) would influence performance, and whether observing several targets move without trying to tap them would improve subsequent attempts to hit targets moving in the same manner.

## Methods

**Figure 1.**
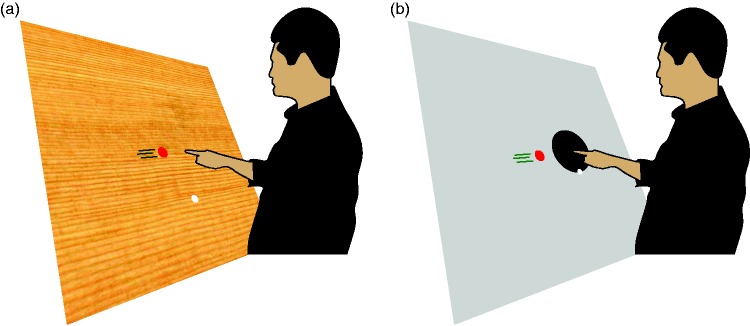
The participant stood in front of a large screen and tried to hit rightward moving targets (shown in red) with their index finger. The targets appeared some time after the index finger was placed at a starting point (shown in white). (a) In Experiment 1, they could hit the target wherever they wanted. The background was one of three photographs (here that of a wooden surface). (b) In Experiment 2, they had to hit the target within an indicated region of the background (within a black disk on a grey background).

The study consists of two experiments. In both experiments, people were asked to tap on targets (simulated disks) that moved rightwards across a large acrylic rear-projection screen (Techplex 150; 1.25 m wide, 1 m high, tilted backwards by 30°). Images (800 × 600 pixels) were presented using an InFocus DepthQ Projector. They were presented at 60 Hz in Experiment 1 and at 120 Hz in Experiment 2. Participants stood in front of the screen in a room with normal fluorescent office illumination. They tapped the screen with their right index finger (Figure 1). They were not restrained in any way. An Optotrak 3020 that was placed at about shoulder height to the left of the screen measured the position of an infrared light-emitting diode attached to the nail of the participant’s right index finger and determined when a second diode was inactivated (both at 500 Hz). The second diode was inactivated for about 10 ms, 1 ms after a flash of light fell on a sensor that was placed in the path of the light directed toward the top left corner of the screen. This allowed us to synchronize the timing of the display with that of the Optotrak precisely enough to be able to determine the position of the finger with respect to the images on the screen with a resolution of 2 ms (for more details, see Brenner & Smeets, 2015).

In both experiments, the participants’ task was to tap on as many targets as possible. They could rest whenever they liked by simply not moving to the starting point (see Figure 1). Before they started, we related positions of the marker attached to the nail of their index finger to corresponding finger positions on the screen by asking the participant to place his or her finger on four, 1 cm diameter red disks. By calibrating finger positions in this manner, we account for the fact that the participant taps with the fingertip rather than the nail (assuming that the orientation of the finger is the same during calibration as during the experiment). After that, new targets appeared between 500 and 800 ms after the participant placed his or her index finger at the starting point (and kept it there until the target appeared). When a target appeared, participants were required to lift their finger off the screen and tap on the target. Taps were detected by the acceleration orthogonal to the screen exceeding 250 m/s^2^ while the finger was less than 5 mm from the screen and less than 10 cm from the target.

Feedback on performance was provided after every trial. If the finger hit the target (i.e., if the calibrated finger position was within the target), the latter remained visible for 500 ms at the (interpolated) position at which it was hit and a tone indicated success (in Experiment 2, the tone was only presented if the target was hit within an indicated region). If the tap missed the target, the target was deflected away from the finger at 100 cm/s, providing precise information about the direction of the error. After the last target, participants saw their score (the number of targets that they had hit) in a high-score list. Participants enjoy the game-like aspect that this introduces, but it is otherwise totally irrelevant.

In both experiments, we were primarily interested in horizontal errors because ignoring the acceleration will give rise to systematic errors in the direction of target motion. We therefore concentrate on such errors. We used the median of these errors to avoid having to make arbitrary decisions about excluding outliers and about dealing with changes in performance due to training or fatigue. We compared the median horizontal errors across conditions, compared them with the errors that we would expect if participants ignored the acceleration, and used a simple model to evaluate how participants learnt from their errors.

### Experiment 1

A total of 36 adults (19 female, 17 male) who did not know the purpose of the experiment and had no obvious visual or motor impairments participated voluntarily in the experiment after signing an informed consent form. The number of participants was determined on the basis of previous experience. The participants were divided into three groups and each performed one of three conditions in a single session. The experiment complied with the faculty ethical guidelines.

In all three conditions, the targets were red, had a diameter of 3 cm, and moved to the right. They appeared 10 cm above and either 25 or 45 cm to the left of the screen center. Three coefficients of friction were simulated, giving rise to target decelerations of 0, 20, and 40 cm/s^2^. For each level of deceleration, we had two initial target speeds, respectively, 60 and 70, 70 and 80, and 80 and 90 cm/s. The starting point was a white, 2-cm diameter disk, 5 cm to the right of, and 15 cm below the screen center. It appeared as soon as the feedback of the previous trial ended and disappeared when the target appeared.

In two of the three conditions, trials with the three target decelerations were randomly interleaved. The only difference between the two conditions was that in the *same image* condition the background was always the same picture of a wooden surface, whereas in the *different image* condition there were three different pictures, each associated with a target deceleration. The pictures were chosen to match the decelerations (or simulated coefficients of friction): an ice surface for no deceleration (0 cm/s^2^), the above-mentioned wooden surface for the deceleration of 20 cm/s^2^, and a plane of sand for the deceleration of 40 cm/s^2^. The picture was visible from the moment the starting point appeared. In the third condition (*separate blocks*), targets with the three values of deceleration (associated with the same background pictures as in the *different image* condition) were each presented in an independent block of trials, with a short break (several minutes) between such blocks. The order of these blocks was counterbalanced across participants.

In the *same image* condition, participants could only account for the deceleration by judging it during the trial. We therefore expected them to make systematic deceleration-dependent errors, as participants had done in the previous study in which we varied the acceleration (Brenner & Smeets, 2015). In the *different image* condition, participants could use the contextual information provided by the background image to anticipate a certain deceleration. In the *separate blocks* condition, participants could compensate for errors on the previous trial by aiming differently on the next trial, as we previously proposed that they do when provided with feedback (Brenner et al., 2013).

There were 201 trials in each condition. In the *separate blocks* condition, these trials were divided into three blocks of 67 trials (each with a different deceleration). We determined the median horizontal error for each value of acceleration in each condition, irrespective of the initial speed and starting point, and conducted an analysis of variance (ANOVA) with condition (*same image*, *different image*, *separate blocks*) as a between-participant parameter and acceleration as a within-participant parameter. Having seen the results, six of the authors (three female, three male) also performed the *different image* condition, to examine whether the information provided by the pictures is more effective if one is fully aware in advance of how it could be used. This group was not included in the statistical analysis.

### Experiment 2

A total of 29 adults (20 female, 9 male) who did not know the purpose of the experiment and had no obvious visual or motor impairments participated voluntarily in this experiment after signing an informed consent form. The number of participants was determined by availability within the time for which we had booked the lab and equipment. The experiment complied with the faculty ethical guidelines. Each participant took part in two identical sessions of 192 trials, with an approximately 5-minute break between the sessions.

The targets had a diameter of 3.5 cm and moved to the right. They appeared 5 cm above and 40 cm to the left of the screen center. They were usually green, in which case they were to be hit, but occasionally they were red, in which case the participant was not to hit them. They were to be hit within an indicated hitting region: a 20-cm diameter black disk, with its center 80 cm to the right of where the targets appeared (see Figure 1(b)). There were accelerating and decelerating targets, each starting at three different speeds. The speeds were selected so that the target would reach the center of the hitting region 600, 700, or 800 ms after it appeared. The finger’s starting point was a white, 2-cm diameter disk, 10 cm below the center of the hitting region (i.e., at the bottom of that region).

Each session consisted of four repetitions of four sequences of 12 trials. The four sequences differed in two respects. In two sequences, the target was always accelerating at 100 cm/s^2^. In the other two, it was decelerating at 100 cm/s^2^. In one sequence with accelerating targets and one with decelerating targets, all targets were green and were to be hit (*immediate* condition). In the other two sequences, the first three targets were red and were not to be hit, while the remaining nine targets were green and were to be hit (*preview* condition). Within each sequence, four of the targets reached the interception region after each of the three possible times. The times were presented in random order. Sequences of trials with accelerating targets alternated with ones with decelerating targets. The first sequence of targets in each session had 12 green accelerating targets. For 16 of the participants, this was followed by a sequence with 12 green decelerating targets (and then the 2 sequences that had red targets). For the remaining 13 participants, the first sequence was followed by 3 red decelerating targets and then 9 green ones (and then the other 2 sequences). Before the actual experiment, participants performed a practice session with 24 targets that moved at one of 3 constant velocities (60, 79, and 103 cm/s, in random order). The 7th, 8th, 9th, 16th, 17th, and 18th targets were red, to practice refraining from hitting red targets.

Whenever a participant tapped on a red target, the whole sequence of 12 trials was excluded from further analysis. For each kind of sequence, we determined the median horizontal error across trials with the same position in the sequence. We did so for each participant separately, and then determined the mean and standard error across participants. We conducted two repeated measures ANOVAs, with factors condition (*immediate* or *preview*) and acceleration (−100 or 100 cm/s^2^). One analysis compared the first tapped target in each sequence, comparing the first target in sequences without red targets with the fourth in sequences with red targets. The second analysis compared the fourth presented targets of all sequences. Obviously, in both cases, we expect to see an influence of acceleration. If observing a target has no influence on subsequent errors, we expect to see no interaction between condition and acceleration when comparing the first tapped targets, and to see a clear interaction when comparing the fourth taps. If observing the motion is just as good as tapping, we expect to see the opposite pattern.

We also fit a simple learning model to the changes in error within the sequence. We assumed that there is some initial judgment error (***x*_1_**) with respect to some offset (***a***), and that the judgment error is reduced by some proportion (***b***) of its value after each trial. Thus, the measured error on trial *t* (***e_t_***) is given by

(1) $$e_{t}=x_{t}+a$$

with

(2) $$x_{t}={(1-b)x}_{t-1}$$

We fit this model to each participant’s data for each kind of sequence (minimizing the squared residuals) and compared the fit parameters across conditions and accelerations with repeated measures ANOVAs.

## Results

### Experiment 1

For 1 of the 12 participants of the *separate blocks* condition, the intermediate deceleration block terminated after 38 of the 67 trials for some unknown reason. This participant’s median values for that condition are therefore based on fewer trials. Otherwise, only trials in which the participant did not try to tap on the target, or tapped too gently, were excluded from analysis (about seven trials per participant). The median time between the target appearing and the participants’ fingers tapping the screen was about 613 ms and did not differ significantly between the conditions, *F* 2, 33 = 1.0, *p* = .4.

**Figure 2.**
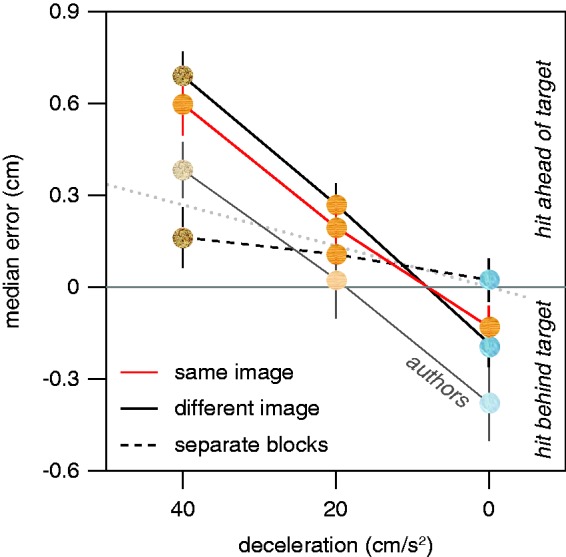
Median horizontal errors in Experiment 1. The three different amounts of deceleration (simulated friction) were interleaved at random with the target always moving across a wooden surface (*same image*), interleaved at random with the target moving across a surface of ice when the deceleration was 0 cm/s^2^, across a wooden surface when the deceleration was 20 cm/s^2^, and across a plane of sand when the deceleration was 40 cm/s^2^ (*different image*), or presented in separate blocks of trials for each deceleration with its associated image (*separate blocks*). The faint symbols represent additional results for six authors in the *different image* condition. The dotted line shows the errors that would arise from not considering the acceleration during the last 116 ms.

The analysis of the median horizontal errors revealed a significant effect of target acceleration, *F*  2, 66 = 72.5, *p* < .0001, and no significant effect of condition, *F* 2, 33 = 1.3, *p* = .28. The effect of target acceleration differed significantly between the conditions (interaction: *F* 4, 66 = 11.7, *p* < .0001). It is evident from Figure 2 that whenever the accelerations were randomly interleaved, participants hit ahead of the targets that decelerated the most and behind the targets that did not decelerate. When the different accelerations were presented in separate blocks of trials, this tendency was very much reduced.

Participants were free to hit the screen whenever and wherever they liked, so we expected the errors in the condition in which the acceleration could not be anticipated (*same image*) to reflect a sensorimotor delay of about 116 ms (Brenner & Smeets, 2015). Not considering a difference in acceleration of 20 cm/s^2^ during the last 116 ms would lead to an error of about 0.13 cm ($\frac{1}{2}\times 20\times 0.116^{2}$). The differences that we found between the median horizontal errors for differences in acceleration of 20 cm/s^2^ were a bit more than twice as large (Figure 2), despite the differences in acceleration being similar to those of the previous study (Brenner & Smeets, 2015).

On average, the authors tapped less far ahead of the targets than did the naïve participants, but their errors depended to a similar extent on the deceleration (similar slopes in Figure 2). Thus, it appears that people readily learn to cope with a single deceleration to which they are repeatedly exposed, but not to interleaved decelerations that are each associated with a different background texture. In Experiment 2, we took a closer look at such learning.

### Experiment 2

One participant stopped half way through the second session, providing us with six rather than eight repetitions of each kind of sequence. We had to remove 14 sequences (1 sequence for two participants, and 2, 4, and 6 sequences for one participant each) because the participants tapped on a red target. Trials in which the participant did not try to tap on a green target, or tapped too gently, were not analyzed, but they were considered when determining the positions of subsequent trials in the sequence (about 21 trials per participant).

**Figure 3.**
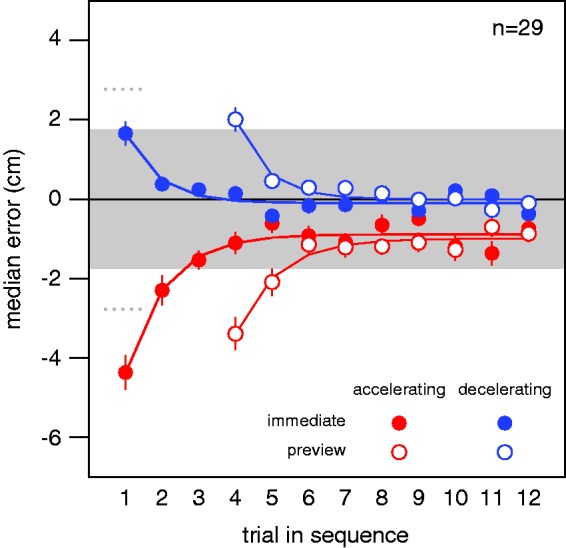
Median horizontal errors in Experiment 2. Error as a function of the trial’s position within the sequence of 12 trials with the same condition and acceleration. Means and standard errors of the participants’ median horizontal errors. The grey area indicates the target’s maximal extent. The curves are fits of the simple learning model to the mean data. The dotted lines at Trial 1 show the errors one would expect if participants used the acceleration during the previous sequence to predict the target’s displacement during the last 169 ms.

Since we indicated where participants should hit the screen, we expected the errors to reflect a sensorimotor delay of about 169 ms (Brenner & Smeets, 2015). Ignoring an acceleration of 100 cm/s^2^ during this time would lead to an error of 1.4 cm ($\frac{1}{2}\times 100\times 0.169^{2}$). If participants learn to compensate for the acceleration, they will be switching from an anticipated acceleration of 100 m/s^2^ to an actual one of  −100 m/s^2^, and vice versa, so the initial error (at least when not first observing red targets) will be twice as large (i.e., 2.8 cm). The sign of the error obviously depends on whether the switch is from accelerating to decelerating, or from decelerating to accelerating. On average, the median horizontal errors were quite close to this value (Figure 3), but there was also an overall tendency to hit behind the target (negative errors).

The ANOVA for the median horizontal error on the first tap in each sequence (solid symbols at Trial 1 and open symbols at Trial 4 in Figure 3) obviously revealed a significant influence of acceleration, *F* 1, 28 = 117, *p* < .0001. It also revealed a significant influence of condition (i.e., of having observed the three red targets), *F* 1, 28 = 7.1, *p* = .01, but no significant interaction, *F* 1, 28  = 1.2, *p* = .3. The influence of condition is visible in Figure 3 as the first taps in the *preview* condition (open symbols at Trial 4) having larger values than the corresponding taps in the *immediate* condition (solid symbols at Trial 1). This means that participants hit further to the right with respect to the target after having observed the three red targets, irrespective of the acceleration. Importantly, the errors were not systematically closer to zero after having observed three targets with the new acceleration without tapping them (there was no significant interaction). The ANOVA for the median horizontal error on the fourth trial of each sequence revealed a significant influence of acceleration, *F* 1, 28 = 62, *p* < .0001 and a significant interaction, *F* 1, 28 = 64, *p* < .0001, with no significant influence of having observed the three red targets, *F* 1, 28 = 0.7, *p* = .4, confirming that observing the targets does not improve subsequent taps in the way that attempting to tap on them does.

The curves in Figure 3 show fits of our simple learning model to the mean of all participants’ median horizontal errors for each kind of sequence. We also fit the model to individual participants’ median horizontal errors and compared these fits’ parameters across conditions and accelerations. For the *immediate* condition, the mean values of the participants’ fit values of ***a***, ***b***, and ***x*_1_** were −0.12, 0.60, and 1.77 for the decelerating targets and −0.39, 0.62, and −4.05 for the accelerating targets. For the *preview* condition, the values were −0.39, 0.58, and 2.46 for the decelerating targets and −0.29, 0.61, and −3.19 for the accelerating targets. Repeated measures ANOVA on these fit parameters revealed no significant differences for either the asymptotic value ***a*** (condition: *F* 1, 28 = 0.07, *p* = .8; acceleration: *F* 1, 28 = 0.07, *p* = .8; interaction: *F* 1, 28 = 0.53, *p* = .5) or the learning rate ***b*** (condition: *F* 1, 28 = 0.13, *p* = .7; acceleration: *F* 1, 28 = 0.20, *p* = .7; interaction: *F* 1, 28 = 0.02, *p* = .9). The ANOVA on the fit initial judgment errors (***x*_1_**) confirmed the previous analysis on the measured initial median horizontal errors: a significant influence of condition, *F* 1, 28 = 4.6, *p* = .04, and of acceleration, *F* 1, 28 = 135, *p* < .0001, but no significant interaction, *F* 1, 28 = .06, *p* = .8. The fit values of ***b*** of about 0.6 mean that the initial systematic errors in each new sequence are reduced to about 40% of their values (with respect to the asymptotic level) on the next trial, about 16% on the third trial, 6% on the fourth trial, and so on (Equation 2; curves in Figure 3). There was a tendency to hit too late (negative values of ***a***). The average fit value for the initial error on each new sequence (absolute value of ***x*_1_**) is about 2.9, which is close to our prediction of an initial error of 2.8 cm.

**Figure 4.**
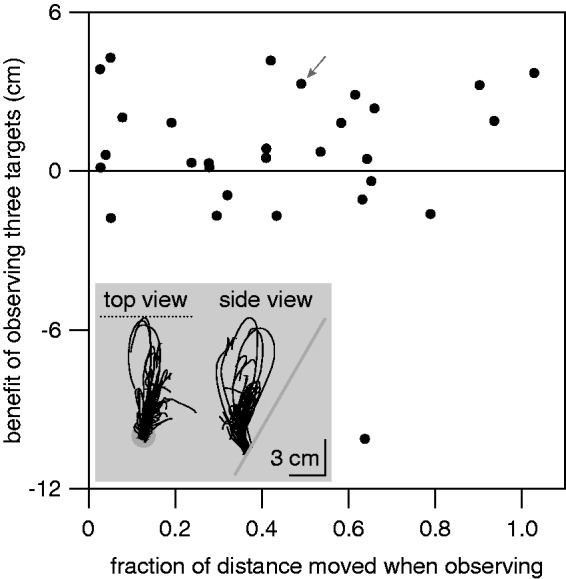
Participants who moved more did not benefit more from the red targets. The inset shows a top and side view of all of one participant’s finger movements during the first 800 ms after red targets appeared. These traces are for the participant indicated by the arrow in the main panel. In the top view (movements parallel to the screen), the grey disk represents the starting point and the dotted line indicates the target’s path. In the side view, the grey line represents the screen surface.

Since observing the target move three times with the new acceleration before trying to hit it did not reduce the median horizontal error, one might wonder whether participants simply ignored the red target’s motion altogether. If they ignored its motion, such motion could not be expected to improve subsequent performance. To examine whether this could be the case, we examined to what extent participants moved toward the red target before realizing that they were not to respond (see inset in Figure 4). In particular, we examined whether despite there being no overall significant benefit of seeing the red targets when considering all the participants, individual participants who moved further toward the target before suppressing their movement might have benefited from “observing” the target.

For each participant, we determined two values. For each sequence of trials in the *preview* condition, we determined the maximal distance that the finger moved up the screen during the three red-target trials, as a fraction of the distance that it moved up the screen on the fourth trial (we call the median of these values the *fraction of distance moved when observing*). We related this to the extent to which the difference between the median horizontal errors for accelerating and decelerating targets was smaller for the fourth trial of the *preview* condition than for the first trial of the *immediate* condition (we call this the *benefit of observing three targets*). If moving the finger toward the target were beneficial, even without receiving the feedback associated with tapping on the target, we would expect to see a positive correlation across participants. The Pearson’s product–moment correlation between these two measures did not suggest that there was any systematic relationship between the amount that the finger moved when the target was red and the extent to which observing the red targets reduced the errors (*r* = −.02; *t*_27_ = −.11, *p* = .9; Figure 4).

## Discussion

In Experiment 2, the median horizontal error on the first tap after each change in acceleration was close to what we predicted on the basis of not considering the target’s acceleration (dotted lines), except that there was also a tendency to tap too late (an overall shift toward negative values). We suspect that the tap is mainly too late, rather than too far to the left, because the average position that was tapped was only 0.07 cm to the left of the center of the indicated interception region, whereas the position of the target at the time of the tap was 0.44 cm to the right of the center of the indicated region (overall averages of median values per participant, condition and acceleration). We can only speculate as to why there was a tendency to tap too late. Perhaps, it was more difficult to speed up when one realized that one was too late than to slow down when one realized that one was too early due to the short time between the target appearing and it reaching the region within which it was to be hit. A reason to think that the tendency to hit behind the targets may be related to having to tap at a certain moment (when the target was within the indicated region) is that we did not observe this tendency in Experiment 1, in which participants could hit whenever they liked. In Experiment 1, there was a tendency to hit too early, as has previously been found when participants could tap on the target wherever they liked (Brenner et al., 2013).

In Experiment 1, we found median horizontal errors that were about twice as large as the systematic errors that we predicted, close to what we would expect for a sensorimotor delay of 169 ms (the delay that we would expect if the interception region were indicated; Brenner & Smeets, 2015). We do not know why. Perhaps, the background image made it more difficult to determine how to adjust the movement. Importantly, the errors were no smaller when the background image indicated the extent of the deceleration (the amount of simulated friction) from before the target appeared (*different image* condition); not even for the authors who knew in advance that the image of sand meant that the target would decelerate, whereas the image of a surface of ice meant that the target would move at a constant velocity. When the three values of deceleration were presented in different blocks of trials, the median horizontal errors were much smaller. Thus, participants readily adjusted their behavior to the targets’ deceleration on preceding trials but did not readily adjust their behavior to the surface across which the target was moving, even though each surface was associated with a different deceleration.

In Experiment 2, we examined how participants adjusted their movements to acceleration on previous trials in more detail. We were particularly interested in whether they had to try to hit the targets, or whether observing how the target moved would be enough to anticipate the next target’s acceleration. Evidently, observing the target move, and even moving toward the target without tapping on the screen, does not help deal with the acceleration on the next trial. The only effect of observing red targets was that participants subsequently tapped a bit earlier, irrespective of the acceleration. Considering that most participants moved a considerable distance when the targets were red (horizontal values in Figure 4), this might be related to a general tendency to hit earlier after every trial in which there is no feedback to inform you that doing so is wrong (Brenner et al., 2013). That observing three targets moving with a new acceleration, and even moving quite far toward these targets without tapping on the screen, did not influence subsequent errors, whereas a single tap reduced the error to about 40% of its initial value, suggests that the feedback associated with the tap is critical.

Could such a mechanism account for earlier findings about how acceleration is used to guide interceptive movements? If we always anticipate the acceleration that we encountered in objects with which we recently *interacted*, the acceleration that we anticipate will shift depending on what we are doing. If we are playing volleyball, we will anticipate gravitational acceleration (and some drag), whereas if we are being handed books to put on a shelf, we will anticipate the deceleration with which the previous books were handed to us. Although even the authors failed to use contextual information to deal with the deceleration in Experiment 1, people might consider a distinction between objects that are likely to be falling under gravitational acceleration and ones that are more likely to be decelerating due to friction, because they do for instance consider the direction from which identical targets appear to approach when timing their actions, even when the conditions are randomly interleaved (Senot et al., 2005). However, this appears to be a crude estimate that needs the kind of adjustments that we saw in Experiment 2 before interception can be accurate and precise.

Adjusting one’s actions in response to feedback, rather than really estimating the acceleration, might seem to be a strange strategy. However, ignoring a constant acceleration during a fixed sensorimotor delay gives rise to an error that is independent of the target’s speed, so using feedback to adjust how far ahead of the target to aim could remove some systematic errors. Since the acceleration of a falling ball is not constant, but depends on the ball’s speed as a result of air resistance (drag; that scales with speed squared), we would expect to see systematic errors if a falling ball’s speed near the moment of contact is varied. We have previously observed systematic errors when the height from which balls were dropped was varied (Brenner, Driesen, & Smeets, 2014), but this was not significant and could not definitively be attributed to changes in acceleration in the way that we could do with virtual targets (Brenner & Smeets, 2015). In order to demonstrate this conclusively with real balls and gravity, we would need to drop balls from a very large range of heights. This example illustrates why such a strategy might actually work. Unless the variations in acceleration are very large, the errors that one makes by ignoring them will be quite modest. Thus, considering large predictable accelerations to some extent (such as that a ball will bounce off a surface or be accelerated by gravity), and further adjusting behavior on the basis of errors when repeatedly performing the same task, might be enough to explain how people can interact with moving targets so proficiently in various experiments as well as in all kinds of daily and sports situations.
